# Multifunctional Nanoparticles in Traumatic Brain Injury: From Targeted Imaging and Diagnosis to Innovative Therapeutics

**DOI:** 10.2147/IJN.S557140

**Published:** 2026-02-05

**Authors:** Nouran Alwisi, Sarah Aqel, Janatul Naeim, Dana Abdulla Al-Hashimi, Samer El Hayek, Firas Kobeissy, Abdullah A Shaito

**Affiliations:** 1 College of Medicine, QU Health, Qatar University, Doha, Qatar; 2 Qatar Biomedical Research Institute, Hamad Bin Khalifa University, Doha, Qatar; 3 American Center for Psychiatry and Neurology, Dubai, United Arab Emirates; 4 Department of Psychiatry, American University of Beirut Medical Center, Beirut, Lebanon; 5 Center for Neurotrauma, Multiomics & Biomarkers, Department of Neurobiology, Neuroscience Institute, Morehouse School of Medicine, Atlanta, GA, USA; 6 Biomedical Research Center (BRC), QU Health, Qatar University, Doha, Qatar; 7 Department of Biomedical Sciences, College of Health Sciences, QU Health, College of Medicine, Qatar University, Doha, Qatar

**Keywords:** traumatic brain injury, nanoparticles, blood-brain barrier, neuroimaging, targeted therapy, exosomes

## Abstract

Traumatic brain injury (TBI) remains a leading cause of morbidity and mortality worldwide, with limited therapeutic progress due to challenges such as impermeability of the blood–brain barrier (BBB) and the multifactorial nature of secondary neurodegeneration. Nanoparticle-based platforms, owing to their tunable physicochemical properties, surface modifiability, and multifunctionality, have emerged as promising tools for both diagnosis and therapy. A wide range of inorganic, organic, and carbon-based nanoparticles has demonstrated improved imaging contrast, enhanced biosensing capabilities, and potential for targeted, real-time diagnostics. On the therapeutic front, nanoparticles have shown the ability to concentrate therapeutic agents at or near injury sites; however, achieving precise delivery remains a major challenge. Indeed, nanoparticle-based therapies are still limited by off-target accumulation in peripheral organs, incomplete BBB penetration, and heterogeneous tissue distribution. Addressing these barriers requires optimizing particle size, surface charge, ligand conjugation, and degradability to improve site-specific targeting and minimize systemic toxicity. In this review, we examine major classes of nanoparticles, including organic, inorganic, carbon-based, and biologically derived nanocarriers, and discuss the key physicochemical properties governing their interactions with the central nervous system. We evaluate their applications in TBI diagnosis, neuroimaging, and therapy, emphasizing the design principles influencing blood–brain barrier penetration, targeting specificity, biodistribution, and clearance. We further assess emerging nanoparticle-based strategies to improve site-specific delivery and mitigate secondary brain injury, and highlight key translational challenges and future clinical directions. Continued research into biodegradable, biomimetic, and environmentally sustainable synthesis methods is essential to advancing nanoparticle design and ensuring their safe and effective integration into the clinical management of TBI.

## Introduction

Traumatic brain injury (TBI) represents a leading cause of disability and mortality across the globe, with annual cases estimated between 27 and 69 million.^1^ Over the past ten years, there has been a steady rise in the number of emergency room visits and fatalities linked to TBI, with typical sources of injury including assaults, motor vehicle crashes, and unintentional falls.^2^ The proportion of TBI cases is almost three times higher in low- and middle-income countries (LMICs) than in high-income countries (HICs).^3^ TBI inflicts not only severe physical harm but also profoundly affects patients’ psychological well-being. It accounts for a significant incidence of cognitive and behavioral impairments in young adults and leaves many survivors with persistent neurobehavioral disabilities.^4^ Clinicians stratify injury severity using the Glasgow Coma Scale (GCS), with scores of 13–15 considered mild, 9–12 moderate, and ≤8 severe. They monitor progression with serial GCS assessments.^5^ Neuroimaging classifications, such as the Marshall CT score and Rotterdam score, further categorize patients based on hemorrhage type, midline shift, and basal cistern status, providing prognostic insight.^5^ Long-term outcomes are often reported using the Glasgow Outcome Scale (GOS) or its extended version, the Glasgow Outcome Scale-Extended (GOSE). Several approaches have been implemented to categorize and guide TBI classification using biomarkers, clinical outcomes, and imaging tools.^6–8^ Several of these markers were based on omics-based approaches that resulted in the FDA-approved TBI biomarkers UCH-L1 and GFAP.^9–16^

**Figure 1:**
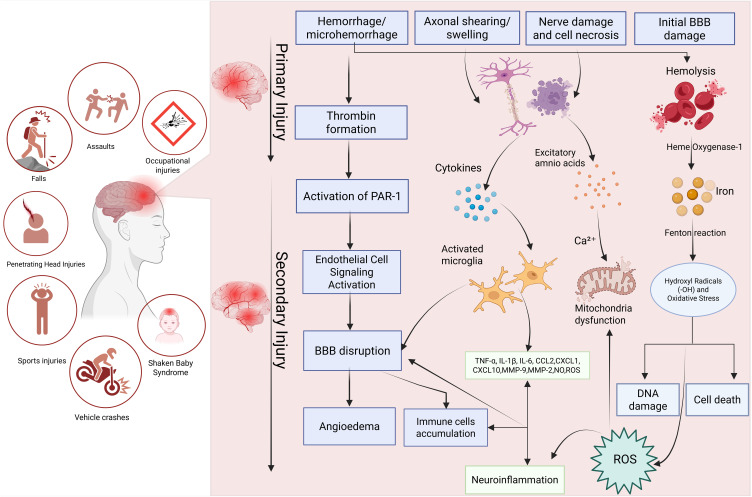
Pathophysiological Mechanisms of Primary and Secondary Injury in TBI. The diagram highlights the complex interplay between vascular, cellular, and molecular processes that contribute to TBI pathogenesis and poor neurological outcomes. On the left, common causes of TBI, such as falls, assaults, occupational injuries, penetrating head injuries, sports injuries, vehicle crashes, and shaken baby syndrome, are shown. The primary injury phase (top right) includes immediate effects such as hemorrhage, axonal shearing, nerve cell necrosis, and initial BBB disruption. These acute events trigger secondary injury mechanisms, including thrombin formation, endothelial cell activation, cytokine release, microglial activation, excitotoxicity, calcium overload, mitochondrial dysfunction, iron-mediated oxidative stress, and further BBB disruption. The resulting cascade leads to neuroinflammation, angioedema, the generation of reactive oxygen species (ROS), DNA damage, and, ultimately, cell death.

Pathophysiologically, TBI includes both primary and secondary injuries. Primary injuries are caused by mechanical stresses, such as rotational shear forces that result in diffuse axonal injury, while secondary injuries arise from oxidative stress, inflammatory cascades, and dysregulation of cerebral blood flow.^17^ These secondary injuries can evolve over hours to days, significantly amplifying the initial damage and complicating the clinical trajectory of affected individuals.^18^ These mechanisms often result in extensive neurological malfunction and cognitive deficits, emphasizing the urgent need for novel therapeutic techniques as the outcomes for TBI patients are still not ideal, even with improvements in acute stabilization, neuroimaging, and surgical procedures, as shown in Figure 1.^19^

Due to the lack of an FDA-approved drug capable of preventing the progression of TBI-induced neurological damage,^20^ acute clinical management of TBI remains primarily supportive. Current interventions focus on maintaining adequate cerebral perfusion pressure, controlling intracranial pressure (ICP) using agents like hyperosmolar solutions (eg, mannitol or hypertonic saline), and performing surgical interventions when necessary. Although these approaches are critical for saving lives and preventing immediate complications, they largely address physiological symptoms rather than the underlying cellular and molecular damage, often failing to improve long-term functional outcomes.^21^ Despite multiple large-scale clinical trials showing that promising neuroprotective agents, previously validated in preclinical TBI models, including corticosteroids, progesterone, and targeted anti-inflammatory compounds, fail to produce significant efficacy in human TBI patients,^22^,^23^ the search for novel and clinically effective TBI therapies remains ongoing. This clinical translational setback underscores the need for fundamentally different approaches to drug delivery and therapeutic targeting.

To address these issues, deeper insights into the pathophysiology of TBI and the development of targeted therapeutics with the capability of penetrating the BBB are needed. Hence, nanoparticle-based therapies, which have become a promising field of study, may have implications for the treatment of TBI.^2^ Due to their small size (less than 100 nanometers), nanoparticles exhibit unique characteristics that distinguish them from bulk materials. They are helpful in many scientific and medical domains due to their small size and high surface-area-to-volume ratio, which provides improved reactivity and adaptability. Unlike bigger particles, nanoparticles can have their form, charge, and stability carefully regulated during synthesis, as well as other physical and chemical properties. Due to their versatility, nanoparticles are now widely used in tissue engineering, drug delivery, and diagnostics, making them a key component of contemporary nanomedicine.^24^ Recent advances in nanotechnology have facilitated the development of multifunctional nanoparticles capable of targeted delivery, real-time imaging, and controlled drug release, offering a promising approach to modulate the complex pathophysiological processes of TBI.^25^,^26^ In this review, we will first explain the different types of nanoparticles and their key properties, then examine how they enhance TBI diagnosis through improved imaging and finally explore their use in delivering treatments directly to injured brain tissue.

## Nanoparticles: Definitions and Types

**Figure 2:**
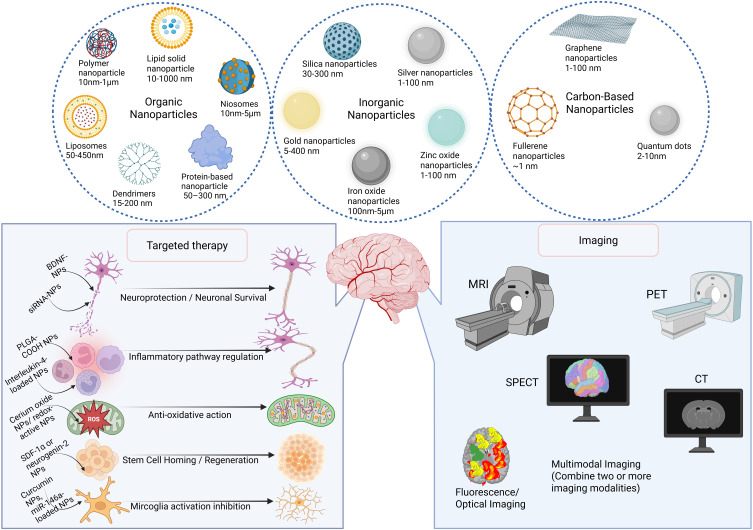
Classes, Therapeutic Applications, and Imaging Modalities of Nanoparticles in Neurotrauma. This figure provides an overview of the main classes of nanoparticles, categorized as organic (eg, liposomes, dendrimers, protein- and polymer-based, lipid solid nanoparticles, niosomes), inorganic (eg, iron oxide, gold, silver, silica, and zinc oxide nanoparticles), and carbon-based nanoparticles (eg, graphene, fullerenes, and quantum dots). The lower left panel illustrates the therapeutic mechanisms of nanoparticles in TBI, including neuroprotection, regulation of the inflammatory pathway, anti-oxidative action, stem cell homing/regeneration, and inhibition of microglia activation. The right panel depicts neuroimaging modalities where nanoparticles serve as contrast agents or probes, including MRI, PET, SPECT, CT, fluorescence/optical imaging, and multimodal imaging approaches. These advances highlight the versatile role of nanoparticles in both therapy and diagnosis of brain injury and neurological disorders.

Nanoparticles (NPs) are materials between 1 and 1000 nm in size whose high surface-area-to-volume ratio, tunable surface chemistry, and ability to cross biological barriers make them powerful tools for targeted drug delivery, imaging, and immunomodulation in TBI (Figure 2).^27^ Broadly, NPs fall into three material classes: organic, inorganic, and carbon-based, with each class encompassing multiple structural motifs (eg, liposomes, dendrimers, nanoshells) that confer distinct advantages and challenges (Table 1).

**Table 1:** Classification, Properties, and Applications of Nanoparticles

Class	Nanoparticle Subtype/Example	Size Range	Key Functions/Applications	Advantages	Limitations/Concerns	References
Organic	Liposomes, SLNs, NLCs (Solid Lipid/Nanostructured Lipid Carriers)	40 nm–1 µm	Drug/gene delivery, BBB crossing, theranostics	Biocompatible, low toxicity, hydrophilic/lipophilic cargo, surface modifiable	Low drug loading (SLNs), instability, rapid clearance	[27–29]
	Polymeric NPs (PLGA, PACA, PCL, Chitosan)	10–1000 nm	Drug/gene/protein delivery, gene therapy, controlled release	Biodegradable, tunable, surface functionalizable, stable	Complex synthesis, burst release, stability issues	[27,28,30]
	Dendrimers (PAMAM, PPI, Glyco, PEGylated)	5–20 nm	Targeted drug/gene delivery, imaging, immune modulation	Highly branched, modifiable, BBB crossing, encapsulation, or surface binding	Synthesis complexity, toxicity (depends on surface), clearance issues	[31,32]
	Micelles (Polymeric/Protein micelles)	10–100 nm	Solubilize hydrophobic drugs, BBB crossing, neuroprotection	Amphiphilic, controlled release, protects cargo	Instability in vivo, premature release	[33,34]
	Protein-Based NPs (Albumin, Gelatin, Collagen)	50–300 nm	Growth factor or drug delivery, sustained release	Natural, biodegradable, multiple drug-binding sites	Possible immunogenicity, lower stability	[27,28]
Inorganic	Metallic NPs (FeONPs, AuNPs, AgNPs, Gd-based NPs, CeO_2_, ZnONPs, PtNPs)	10–100 nm	MRI, CT, PET imaging, magnetic targeting, neuroprotection	Easy to functionalize, tunable size, imaging/therapy (theranostics)	Potential neurotoxicity, tissue accumulation	[35, 36]
	Quantum Dots (QD, SiQDs, *AgInSe_2_QD)*	2–10 nm (QDs)	NIR/fluorescent imaging, BBB crossing, protein aggregation inhibition, ROS scavenging	Bright, stable, sensitive, BBB crossing, targeted imaging	Neurotoxicity, tissue accumulation, oxidative stress	[27,28,37]
	Nanoshells & Nanocapsules (Gold Nanoshells, Silica Nanocapsules)	~50–900 nm	Photothermal therapy, multimodal imaging, triggered drug release	Versatile, customizable, can load various cargoes	Stability, potential toxicity	[38,39]
Carbon-Based	Graphene & Graphene Oxide (GO)	~1–100 nm	Drug/gene delivery, photothermal therapy, ROS scavenging	High surface area, tunable, large drug loading, photothermal	Aggregation, toxicity, costly production	[40–42]
	Fullerenes (C_6__0_)	~1 nm	Antioxidant, photodynamic therapy, carrier for drugs	Free-radical scavenger, biocompatibility (after modification), photodynamic action	Hydrophobicity (unless modified), potential toxicity	[27,28,43]
	Carbon Quantum Dots (CQDs, GOQDs, Graphene QDs)	<10 nm (CQDs)	Bioimaging, drug delivery, antioxidant therapy	Biocompatible, multicolor imaging, ROS scavenging	Limited clinical data, possible cytotoxicity	[27,28,44]
	Carbon Nanotubes (SWCNT/MWCNT)	~1–100 nm (diameter)	Drug/gene delivery, neural interfaces, biosensing	Strong, electrically active, large surface area	Water insoluble, toxicity, inflammation risk	[27,28,45,46]

**Notes**: Table 1. Summary of nanoparticles, Organic: Composed mainly of lipids, polymers, or proteins; known for biocompatibility and versatility in drug delivery. Inorganic: Include metals and metal oxides; excel in imaging, targeting, and combined therapy/imaging (theranostics). Carbon-Based: Include graphene, fullerenes, carbon dots, and nanotubes; notable for unique electrical, antioxidant, or photothermal properties but may need modification for biocompatibility.

### Organic Nanoparticles

#### Lipid-Based NPs

Liposomes (50 nm–1 µm) are phospholipid bilayers that encapsulate an aqueous core loading both water- and lipid-soluble drugs. PEGylation extends circulation, and peptide ligands confer lesion homing.^47^ Solid lipid nanoparticles (SLNs) and nanostructured lipid carriers (NLCs) represent advanced lipid-based drug delivery systems, ranging in size from 40 to 1000 nm.^48^ SLNs contain solid lipid cores stabilized by surfactants, making them suitable for encapsulating both hydrophilic and hydrophobic drugs. This provides stability, controlled release, biodegradability, and ease of production. However, their highly crystalline lipid structure limits drug loading and can lead to payload expulsion during storage. To overcome these limitations, NLCs integrate mixtures of solid and liquid lipids, forming less ordered matrices that enhance the drug loading capacity and minimize expulsion.^48^

#### Polymeric NPs

Polyester NPs (PLGA; 100–1000 nm) are FDA-approved, biodegradable, encapsulates small molecules, peptides, and nucleic acids. Surface ligands target astrocytes, microglia, and endothelial cells.^49^

- Dendrimers (5–20 nm) are highly branched polymers with multivalent end groups; they are efficiently endocytosed by microglia and immune cells for siRNA or drug delivery.^50^
- Micelles (10–100 nm) are amphiphilic block copolymer assemblies; hydrophobic cores solubilize lipophilic drugs, extravasate across leaky BBB to deliver neuroprotective agents.^51^
- Polymeric nanorods (tens–hundreds nm in length) have a high aspect ratio, which enhance vascular margination and prolong circulation. They are used for targeted delivery to endothelial and immune cells.^52^

#### Protein-Based NPs

- Albumin NPs (50–300 nm) are a natural carrier with multiple drug-binding sites, exemplified by Abraxane™ in oncology, with potential for neurotrophic factor delivery.^27^
- Collagen/Gelatin NPs are biodegradable matrices for sustained release of growth factors or small drugs.^27^

### Inorganic Nanoparticles

#### Magnetic NPs (MNPs)

Iron oxide nanoparticles (IONPs), including magnetite (Fe_3_O_4_) and its oxidized form maghemite (γ-Fe_2_O_3_), are widely used due to their ability to prevent particle aggregation and maintain stability in biological fluids.^53^

#### Nanoshells & Nanocapsules

Nanoshells and nanocapsules are hollow inorganic structures engineered for multimodal diagnosis and therapy.

- Gold Nanoshells (~50–200 nm) contain an inorganic shell over a dielectric core; photothermal activation for combined imaging/therapy.^54^
- Silica Nanocapsules (50–900 nm) feature a porous or hollow silica shell that can be loaded with gases (for ultrasound or photoacoustic contrast) or therapeutic payloads. Triggered release is achieved via shell degradation, pH change, or external stimuli, making them versatile carriers for on‐demand drug delivery in TBI and other application.^55^

### Carbon-Based Nanoparticles

#### Graphene & Graphene Oxide

Graphene and its oxide derivative, graphene oxide (GO), have emerged as highly versatile platforms for drug delivery owing to their exceptionally large surface area and tunable chemistry.^40^ Their extended π‐conjugated structure allows for high drug loading whether through π–π stacking or covalent attachment. Surface functionalization with targeting ligands (eg, antibodies, peptides) directs nanocarriers to specific cells or tissues. They form composites with metal NPs for enhanced drug loading and photothermal therapy.^27^

#### Fullerenes (C_6__0_)

Spherical cages enable the entrapment and transport of diverse agents, while their extensive surface area promotes strong interactions with biological environments.^43^ Act as powerful free-radical scavengers by accepting multiple electrons into their conjugated cages. Fullerenes serve as photosensitizers in photodynamic therapy by producing reactive oxygen species when illuminated, thereby selectively destroying target cells.^43^ To address fullerenes’ inherent hydrophobicity, researchers have grafted hydrophilic polymers or stimuli-responsive linkers such as ROS-sensitive bonds for on-demand release under oxidative stress or PEG chains for prolonged circulation thereby enhancing solubility, biocompatibility, and targeted delivery while minimizing side effects.^56^

#### Carbon Quantum Dots (CQDs)

CQDs are <10 nm fluorescent nanoparticles that combine excellent biocompatibility with high drug‐loading capacity.^57^ CQDs improve the solubility of hydrophobic drugs and can co-deliver multiple agents for synergistic effects. Preclinical models demonstrate that doxorubicin- and methotrexate-loaded CQDs enhance efficacy and reduce toxicity in cancer.^57^ Additionally, antioxidants loaded into CQDs benefit treatments for eye and brain injuries.

## Mechanisms of Blood–Brain Barrier Penetration by Nanomaterials

The diversity of nanoparticles described above offers a highly adaptable platform for addressing the diagnostic limitations associated with TBI. The collective physicochemical attributes, including tunable particle size, inherent surface modifiability, and unique functional modalities (eg, superparamagnetism in IONPs or intrinsic fluorescence in CQDs), enable these agents to circumvent major biological impediments, most notably BBB permeability. Through strategic optimization of these features, nanoparticles can be engineered to deliver contrast agents selectively to pathological biomarkers, including activated microglia, sites of vascular leakage, or regions characterized by oxidative stress, thereby supporting the development of high-resolution, molecular imaging techniques essential for accurate TBI diagnosis and reliable longitudinal monitoring.

**Figure 3:**
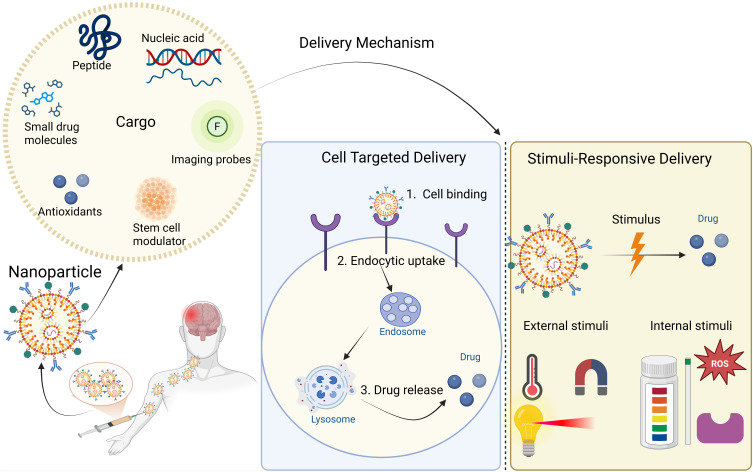
Transport mechanisms across the blood-brain barrier. Schematic illustration of major mechanisms by which nanoparticles cross the blood–brain barrier (BBB): transcellular diffusion (lipophilic drugs), paracellular transport (small hydrophilic molecules), receptor-mediated transcytosis (transferrin, ApoE ligands), cell-mediated transcytosis (macrophages, exosomes), carrier-mediated transport (GLUT1, LAT1 substrates), and adsorptive-mediated transcytosis (cationic nanoparticles).

Nanomaterials can traverse the BBB through several internalization mechanisms that depend on their size, surface chemistry, and functionalization (Figure 3).^58^ The most prominent entry pathway is Receptor-Mediated Transcytosis (RMT), in which surface ligands such as analogs of transferrin, lactoferrin, or apolipoprotein E bind to endothelial receptors to facilitate active vesicular transport into the brain parenchyma.^59^ Adsorptive-Mediated Transcytosis (AMT) is also involved, driven by non-specific electrostatic interactions between cationic nanoparticle surfaces and the negatively charged endothelial membranes.^60^ In addition, smaller lipophilic nanoparticles can passively diffuse across the BBB via simple transcellular diffusion, while paracellular transport is typically limited by tight junction integrity. In addition, emerging evidence suggests that cell-mediated delivery, utilizing peripheral immune cells or naturally secreted extracellular vesicles as biological carriers, may further enhance nanoparticle internalization and transport across the BBB, particularly under inflammatory conditions.^61^

Species-specific BBB properties can undermine the predictive power of pre-clinical nanomedicine studies. Rodent vessels are thinner, have higher trans-endothelial electrical resistance (TEER) and distinct expression of efflux transporters, which often yields exaggerated nanoparticle permeation compared with humans.^62^ Immune surveillance also diverges: mice rely heavily on neutrophil-driven clearance in the liver and spleen, whereas larger mammals exhibit a more balanced monocyte-macrophage response, altering systemic nanoparticle half-life and biodistribution.^58^ Consequently, clearance kinetics observed in rats may overestimate brain exposure and underestimate peripheral toxicity.^58^

Large-animal models (pigs, non-human primates) possess a cerebral vasculature, tight-junction architecture, and immune repertoire that more closely resemble human physiology, providing a more reliable bridge to clinical outcomes. However, they remain costly and ethically demanding, limiting throughput.^63^

Human-derived BBB organoids and organ-on-a-chip platforms recapitulate key cellular components (endothelial cells, pericytes, astrocytic end-feet) and can be tuned to human-like TEER values, offering mechanistic insight into nanoparticle transcytosis and immune interactions without inter-species confounders. Their main limitation is the absence of a full circulatory system, so systemic clearance cannot be modeled directly. An integrated workflow initial screening in organoid/ chip systems followed by validation in large-animal studies maximizes predictive accuracy while balancing practicality and ethical considerations.^64^

## Nanoparticles for the Diagnosis of TBI

### Iron-Oxide-Based Nanoparticles (IONPS)

Magnetic nanoparticles, particularly those composed of iron oxide, significantly enhance Magnetic Resonance Imaging (MRI) sensitivity in the diagnosis of TBI (Table 2). While other types of contrast agents are available, iron-oxide-based nanoparticles (IONPS) have been used as *T*_2_ contrast agents are commonly employed because they are simple to make, functionalize, biocompatible, and cost-efficient.^65^ The introduction of iron-oxide magnetic nanoparticles might improve medical applications, such as cell labeling and sorting, cell transfection, and diagnostic imaging based on MRI, PET, or multimodal imaging. Two iron oxides, magnetite (Fe_3_O_4_) and its oxidized and more stable form, maghemite (γ-Fe_2_O_3_), are commonly used in biomedical applications due to their favorable properties.^66^ These substances exhibit superparamagnetic behavior at diameters less than around 25 nm for magnetite and 30 nm for maghemite. This feature makes them suitable for applications that require contrast enhancement in MRI. Because the biological distribution of nanoparticles is directly proportional to their size, they have been classed as follows: There are three types of iron oxide nanoparticles: ultra-small (USPIONs) with a diameter of less than 50 nm, superparamagnetic (SPIONs) with a diameter of hundreds of nanometers, and micron-sized (MPIO) with a diameter of more than 1 μm.^66^ USPIONs (<50 nm) provide prolonged circulation and deep penetration, making them suitable for diffuse injuries, while MPIOs (>1 μm) have higher magnetic moments and stronger retention, allowing for clearer detection of focal injuries such as microbleeds or vascular lesions.

**Table 2:** Comparison of Nanoparticles for Traumatic Brain Injury Diagnostics

Nanoparticle Type	Mechanism/Key Properties	Imaging Modality	Advantages	Limitations	References
Iron-oxide NPs (IONPs)	Superparamagnetic Fe_3_O_4_ or γ-Fe_2_O_3_ cores; size-tunable (USPION <50 nm for deep penetration, MPIO >1 μm for focal lesions)	T_2_-weighted MRI (± PET, multimodal)	Well-established, biocompatible, cost-efficient; Strong T_2_ contrast; Adjustable circulation and tissue distribution	Potential to induce oxidative stress Limited molecular specificity without ligands	[65–67]
Gold-coated Fe_3_O_4_ (Au-Fe_3_O_4_)	Fe_3_O_4_ core (superparamagnetic) + Au shell (CT contrast, plasmonic); surface functionalization with peptides/antibodies	T_2_ MRI / CT / photoacoustic / fluorescence	Dual MRI + CT contrast; Optical & PA imaging; Customizable active targeting	Complex synthesis Risk of long-term gold retention	[68–70]
FeCo/FePt NPs	FeCo: ultra-high saturation magnetization; FePt: stable L1_0_ crystal; require protective coatings (graphitic, silica, PEG)	T_2_ MRI (± CT, optical, hyperthermia)	Exceptional magnetic moment; Magnetic hyperthermia (FePt); Enhanced stability	Surface reactivity/oxidation Complex crystal growth Limited in vivo safety data	[71,72]
Gadolinium chelates (Gd-DTPA, DOTA, DPDP)	Gd^3^⁺ chelated by linear (DTPA) or macrocyclic (DOTA) ligands; shortens T_1_ relaxation via unpaired electrons	T_1_-weighted MRI	High relaxivity and soft-tissue contrast; FDA-approved macrocyclic agents	Nephrogenic systemic fibrosis risk Rapid clearance requires repeat dosing	[66,73]
Manganese oxides (MnO, MnO_2_, Mn_3_O_4_)	Mn^2^⁺ provides intrinsic T_1_ contrast; enters cells via Ca^2^⁺ channels; formulated as nanoparticles or chelates	T_1_-weighted MRI/ PET	Endogenous metal with metabolic clearance; Strong T_1_ contrast	Neurotoxicity at high accumulation Narrow therapeutic window	[65,74,75]
Stimuli-responsive NPs (pH-, MMP-, ROS-sensitive)	Acid-labile bonds, MMP-cleavable peptide linkers, or ROS-labile moieties (thioketal, peroxalate, boronate)	Fluorescence / MRI / OCT / PA / multimodal	Lesion-specific activation reduces background; High spatiotemporal precision; Enables theranostic release	Trigger heterogeneity Possible off-target activation	[76,77]
Mesoporous Silica Nanoparticles (MSNs)	High-surface-area silica scaffold loaded with dyes/Gd chelates; capped with ROS- or pH-sensitive linkers that “uncap” in lesions	Fluorescence / T_1_ MRI / PET/SPECT	Extremely high payload capacity; Multimodal readouts; Lesion-specific activation; Tunable release profiles	Potential biodegradation products Long-term safety under study	[78,79]
PEG-capped AgInSe_2_ core	Silver indium selenide cores emit in NIR (700–900 nm) via quantum confinement PEG surface resists phagocytosis and aggregation	Near-infrared fluorescence	Deep-tissue, high-contrast imaging under ambient lightUltralow photobleaching suitable for long surgeriesReal-time intraoperative guidanceDifferentiates intracranial vs superficial bleeding	Potential long-term bioaccumulation/toxicity Limited penetration depth vs longer-wave NIRII probes Clearance kinetics need further study Regulatory path for clinical use	[80]
Exosomes	Naturally secreted vesicles (30–150 nm) carrying endogenous proteins/RNAs; can be labeled with imaging probes	MRI / PET / fluorescence / optical	Innate BBB crossing; Biomarker-rich cargo; Highly biocompatible	Isolation/purification challenges Batch heterogeneity Regulatory hurdles	[81,82]

**Notes**: Table 2. This table summarizes key nanoparticle classes used in TBI imaging, detailing their primary mechanisms, compatible imaging modalities, major advantages, and current limitations.

### Gold-Coated Iron Oxide Nanoparticles (Au-Fe3O4)

The Fe_3_O_4_ core exhibits superparamagnetic behavior, enabling enhanced T2-weighted MRI for the detection of tissue damage, hemorrhage, and edema. Simultaneously, the gold shell provides strong X-ray attenuation for CT and serves as a surface for conjugation with fluorescent dyes or near-infrared (NIR) labels, making it compatible with optical imaging modalities such as photoacoustic and fluorescence imaging. This dual-contrast functionality allows Au-Fe_3_O_4_ nanoparticles to bridge high-resolution soft tissue MRI with structural CT and intraoperative optical visualization, providing comprehensive anatomical and molecular characterization of TBI lesions.^83^ Their surface chemistry is easily modifiable with targeting ligands (eg, RGD peptides, CAQK, antibodies against ICAM-1 or TNF-α), enabling selective accumulation in inflamed brain regions or sites of BBB disruption.^84^ Additionally, their plasmonic resonance properties are exploitable for photoacoustic imaging, which facilitates deep-tissue, high-contrast imaging of oxidative stress and neuroinflammation. Some preclinical studies have demonstrated the utility of Au-Fe_3_O_4_ nanoparticles not only for diagnostics but also as theranostic tools, capable of delivering therapeutic payloads while simultaneously monitoring treatment efficacy through real-time imaging.^83^,^85^

### Iron-Cobalt (FeCo) and Iron-Platinum (FePt)

FeCo nanoparticles, in particular, demonstrate 2–3 times greater saturation magnetization than SPIONs, making them potent contrast agents. However, their inherent reactivity and susceptibility to oxidation necessitate surface modification, commonly achieved through the use of graphitic carbon, silica, or PEG coatings to improve biostability and reduce cytotoxicity. FePt nanoparticles, on the other hand, combine magnetic responsiveness with enhanced chemical stability. Their unique L1_0_ crystal structure provides them with strong and stable magnetic properties,^86^ which make them excellent for enhancing T2-weighted MRI contrast.^85^ When their surfaces are modified with the right materials, these nanoparticles can also be used for dual-modality imaging, combining MRI with CT or optical techniques. Moreover, FePt particles can be engineered for magnetic hyperthermia, offering theranostic potential. While their application in TBI-specific clinical imaging is still emerging, these nanoparticles hold considerable promise for high-resolution brain imaging, intraoperative MRI, and longitudinal monitoring of neuroinflammation and neurodegeneration following traumatic injury. Neuroinflammation, which can be triggered by TBI, can also be diagnosed through a multimodal imaging strategy based on PET and MRI probes, as sulphated dextran-coated iron oxide nanoparticles are highly taken up by activated microglia.^73^ Because of their large magnetic moments, some paramagnetic ions, such as dysprosium (Dy3+), have been proposed as viable replacements for iron oxide T2 contrast agents in high-field MRI. Dy3+ has been employed as a chelate (Dy3+-DTPA) and as nanoparticles (Dy2O3).^87^

### Free Gadolinium (Gd) Ions

Free Gd ions are cytotoxic and accumulate in the liver, spleen, and bone. To avoid this toxicity, Gd is chelated, a process that involves large organic molecules forming a stable complex around it. The chelate minimizes the risk of toxicity caused by Gd exposure. The kidneys primarily remove the stable compound. Examples of chelating compounds include diethylene-triamine-pentaacetic acid (DTPA), 1,4,7,10-tetraazacyclo-dodecane-1,4,7,10-tetraacetic acid (DOTA), and dipyridoxyl-di-phosphate (DPDP). Gd-based contrast agents work by shortening the longitudinal relaxation time (T_1_) of nearby protons in the tissue. The gadolinium ion’s unpaired electrons create a local magnetic field, which accelerates the relaxation process of surrounding hydrogen protons. This leads to an increased signal intensity (SI) in T_1_-weighted MRI images, allowing for better visualization of tissues, blood vessels, and abnormalities.^66^ Gd-containing contrast agents are primarily classified into two main types: linear and macrocyclic complexes. Linear Gd complexes have a simpler, linear structure where linear ligands chelate the gadolinium ion. While effective, linear agents are less stable compared to macrocyclic agents, and there is a higher risk of gadolinium ion release, which can lead to toxicity. Macrocyclic Gd complexes, on the other hand, have a more rigid, cyclic structure that provides enhanced stability and a lower risk of gadolinium leakage. This makes them the preferred choice in clinical practice today due to their improved safety profile and effectiveness in preventing gadolinium retention in tissues. Gd-based contrast agents can be highly toxic and are associated with nephrogenic systemic fibrosis. Manganese represents the most viable alternative to gadolinium.^73^

### Manganese Oxides

Manganese oxides, such as MnO, MnO2, and Mn3O4, are used to create these nanoparticulate systems. Although Mn2+ is a normal cellular ingredient similar to Ca2+, excessive quantities of Mn dust are hazardous. Furthermore, given Mn2+’s ability to enter cells via calcium channels, Mn complexes, dendritic Mn chelates, and even Mn nanoparticulate systems have potential applications in neuroimaging. These Mn-based agents enhance MRI by providing strong T_1_ contrast.^65^ However, their use raises concerns about the brain’s sensitivity to Mn exposure, as accumulation could lead to neurotoxicity, emphasizing the need for careful dose management and biocompatibility assessments in the development of Mn-based imaging tools.^65^

### Stimuli-Responsive Nanoparticles

Stimuli-responsive nanoparticles that respond dynamically to changes in the TBI microenvironment are emerging as highly promising agents for enhanced diagnostic imaging. These nanoparticles are engineered to sense and respond to specific physiological cues such as local pH variations, elevated enzyme activities, and oxidative stress markers, which are hallmark features of the TBI milieu.

### pH-Sensitive Nanoparticles

pH-sensitive nanoparticles represent a class of stimuli-responsive nanocarriers designed to exploit the acidic microenvironment that characterizes sites of secondary injury in TBI. Following TBI, the disruption of cerebral blood flow, mitochondrial dysfunction, and the onset of anaerobic glycolysis lead to the accumulation of lactic acid and a subsequent decrease in local pH, often reaching values as low as 6.0–6.8 in the injured tissue.^88^ This acidic milieu provides a biochemical trigger that can be harnessed for site-specific drug release. pH-sensitive nanoparticles are commonly constructed using acid-labile linkers (eg, hydrazone, imine, or acetal bonds) or pH-responsive polymers such as poly(β-amino esters), poly(histidine), or poly(L-histidine)-grafted polyethylene glycol, which undergo conformational changes or degradation in acidic conditions.^89^,^90^ By selectively activating imaging agents only in the acidic regions of injured tissue, pH-sensitive nanoparticles have been incorporated into theranostic platforms by conjugating imaging moieties such as pH-responsive fluorophores or pH-activated MRI contrast agents, allowing for real-time monitoring of drug release and lesion pH.^91^ This allows for more accurate localization and delineation of TBI lesions, minimizing interference from healthy brain regions.

### Matrix Metalloproteinases (MMPs)

MMPs are a family of zinc-dependent endopeptidases that play a pivotal role in extracellular matrix (ECM) remodeling, particularly during inflammatory and repair processes following TBI. In the acute and subacute phases of TBI, elevated expression of MMPs, especially MMP-2, MMP-3, and MMP-9, is observed in both the BBB and surrounding parenchymal tissues.^92^ These enzymes contribute to BBB breakdown, vascular permeability, gliosis, and neuronal degeneration, making them valuable biomarkers of injury progression and therapeutic response.^92^

MMP-sensitive nanoparticles are specifically engineered to contain cleavable peptide sequences or polymeric linkers that are substrates for MMPs.^93^ Upon encountering elevated MMP levels in the TBI microenvironment, these linkers are enzymatically degraded, triggering the site-specific activation of imaging signals.^94^ For instance, this can lead to the release of a quenched fluorescent dye, resulting in localized fluorescence, or the unmasking of MRI contrast agents such as gadolinium or iron oxide cores.^95^,^96^ This strategy enables real-time, non-invasive visualization of pathological ECM remodeling and inflammation, which is particularly useful for differentiating between active and resolving lesions. In addition to improving diagnostic specificity, MMP-activatable nanoparticles can also enhance the spatiotemporal mapping of secondary injury zones, such as areas at risk of expansion or delayed degeneration.^93^ These innovative systems may be adapted for multiple imaging modalities, including fluorescence imaging, optical coherence tomography (OCT), photoacoustic imaging, and MRI, depending on the payload and signal mechanism. Furthermore, MMP-cleavable designs can be incorporated into theranostic platforms, combining diagnostic imaging with the release of therapeutic agents such as anti-inflammatory drugs or antioxidants in response to MMP activity.^97^

### ROS-Responsive Nanoparticles

Following TBI, the imbalance between ROS production and antioxidant defenses leads to oxidative stress, which exacerbates neuronal damage and disrupts the BBB. Nanoparticles engineered with ROS-sensitive chemical moieties are capable of responding to these pathological environments by undergoing structural or functional transformations that activate imaging signals. For instance, cerium oxide (CeO_2_) nanoparticles, owing to their reversible redox cycling between Ce^3^⁺ and Ce^4^⁺ states, function both as ROS scavengers and as ROS-sensitive contrast agents by modulating their catalytic and fluorescence properties in situ.^98^ Boronate ester-modified polymeric nanoparticles exhibit high specificity for hydrogen peroxide (H_2_O_2_), one of the predominant ROS in TBI, cleaving to release quenched fluorophores and thereby enabling precise optical imaging of oxidative microdomains.^99^ In addition, iron oxide nanoparticles conjugated with ROS-labile linkers (eg, thioketals or peroxalate esters) have been developed as activatable MRI probes, remaining magnetically silent under physiological conditions but generating strong T_2_-weighted contrast upon exposure to ROS-rich environments in the injured brain parenchyma.^100^ Ferrocene, a metallocene containing iron sandwiched between two cyclopentadienyl rings, exhibits unique redox properties that enable its oxidation from Fe^2^⁺ to Fe^3^⁺ in ROS-rich microenvironments.^101^ When hydrophobic ferrocene is copolymerized with hydrophilic monomers, it forms amphiphilic block copolymers capable of self-assembling into micellar or vesicular nanostructures in aqueous environments. In the presence of elevated ROS, as observed in TBI lesions, ferrocene moieties undergo oxidative conversion to more hydrophilic ferrocenium species. This redox-triggered polarity shift disrupts the hydrophobic–hydrophilic balance of the copolymer, leading to nanocarrier destabilization, disassembly, and controlled release of encapsulated therapeutic or imaging agents.^101^ Recent advancements include ROS-sensitive liposomes encapsulating gadolinium-based contrast agents; lipid peroxidation in these nanocarriers induces membrane destabilization and controlled release of gadolinium, significantly enhancing lesion detection in preclinical TBI models.^100^

### Mesoporous Silica Nanoparticles (MSNs)

MSNs have emerged as a treatment for TBI, owing to their tunable pore architecture, high surface area, and facile surface chemistry. By encapsulating therapeutic cargos ranging from small-molecule antioxidants to large proteins, such as brain-derived neurotrophic factor (BDNF), within their 2–10 nm pores, MSNs afford controlled, sustained drug release directly at injury sites, thereby mitigating secondary mechanisms such as neuroinflammation and oxidative stress.^102^ Surface grafting of targeting ligands (eg, transferrin or angiopep-2) enables receptor-mediated transcytosis across the BBB, ensuring efficient accumulation in damaged regions. MSNs have been used to target and deliver drugs to amyloid-beta plaques, which are implicated in TB. For example, a recent study engineered 48 nm MSNs functionalized with a fluorescent chalcone “switch” and a DTPA chelator for ^99m^Tc labeling. These particles remained over 90% cell-safe in two cell lines, lit up fourfold, and shifted their emission by 30 nm when they bound Aβ_4__2_ aggregates in vitro. They were also shown by SPECT scans to penetrate the rabbit brain. In a mouse model overexpressing Aβ_4__2_ after repetitive mild TBI, about 4.4% of the injected dose accumulated in the brain at two hours (versus 0.25% in controls) and washed out slowly over hours. When loaded with curcumin, the same MSNs released the drug in two distinct phases, demonstrating their dual capacity for both precise imaging and therapeutic delivery.^102^

### Silver Indium Selenide (AgInSe_2_) Quantum Dots

In this approach, NIR-II-emitting quantum dots (QDs) composed of silver indium selenide cores and an epitaxially grown aluminum‐doped zinc sulfide shell are capped with polyethylene glycol to resist phagocytic clearance and withstand prolonged light exposure and complex biological environments.^80^ When administered intravenously in a mouse TBI model, these QDs enable high‐resolution fluorescence imaging that not only pinpoints the location and extent of primary injury but also distinguishes between superficial and intracranial bleeding in real time. During surgical intervention, the same QDs continuously monitor any secondary hemorrhage and guide neurosurgeons in deciding when and how extensively to evacuate intracranial hematomas.^80^

### Exosomes

Exosome-based imaging is emerging as a promising diagnostic tool in TBI.^103^ Exosomes are small (30–150 nm) naturally derived extracellular vesicles released by various cell types and play critical roles in intercellular communication by transporting proteins, lipids, RNA, and DNA. Their ability to cross the BBB and reflect the physiological and pathological states of their originating cells makes them ideal candidates as both biomarkers and imaging agents for neurological disorders like TBI.^103^ Exosomes isolated from biofluids such as blood, cerebrospinal fluid, or saliva carry injury-specific cargo, including microRNAs and proteins that correlate with the extent and phase of brain injury, thereby providing sensitive and minimally invasive diagnostic insights even at early stages post-injury.^81^ Furthermore, exosomes can be engineered or labeled with various imaging probes such as fluorescent dyes, radionuclides, or magnetic nanoparticles to facilitate real-time, non-invasive tracking of their biodistribution and accumulation in injured brain tissue using modalities like MRI, PET, or fluorescence imaging.^82^ This enables clinicians to not only detect the presence of injury but also monitor inflammatory responses, repair processes, and therapeutic efficacy.^81^ However, challenges remain in standardizing isolation protocols, enhancing targeting specificity, and ensuring safety and reproducibility before clinical application.

Extracellular vesicles (EVs), including exosomes and microvesicles, are increasingly recognized as “natural” nanoparticles for TBI diagnostics.^104^ These membranous nanoparticles (30–1000 nm) can be enriched from patient biofluids and profiled for both their physical properties and molecular cargo. Techniques such as nanoparticle tracking analysis (NTA) or tunable resistive pulse sensing (TRPS) quantify EV size and concentration, providing an indirect measure of injury-induced vesicle release.^104^ Beyond enumeration, immunoaffinity capture using magnetic beads or antibodies against neuronal surface markers (eg, L1CAM) isolates brain-derived EVs for downstream analysis. Proteomic and RNA profiling of these nanoparticles has revealed elevated levels of TBI-relevant biomarkers such as Aβ_4__2_, phosphorylated tau, GFAP, and UCH-L1 that correlate with injury severity, BBB disruption, and temporal progression post-TBI.^104^ Thus, leveraging EVs as endogenous nanoparticle carriers enables minimally invasive, highly sensitive detection of molecular signatures in TBI, offering both acute diagnostic utility and a window into evolving secondary injury cascades.

Astrocyte-derived exosomes (AS-Exos) hold great promise as diagnostic agents for TBI by virtue of their inherent cell-specific cargo and natural ability to cross the BBB.^105^ When released into the circulation following injury, AS-Exos encapsulate astrocyte-derived proteins (eg, GFAP, S100β) and microRNAs (such as miR-21) that faithfully reflect the extent and timing of cerebral insult.^105^ By enriching for exosomes bearing astrocyte markers (eg, GLAST, AQP4), one can achieve high diagnostic specificity and minimize background from other extracellular vesicle populations.^106^ AS-Exos can also be engineered to carry imaging contrast moieties, such as fluorophores, radionuclides, or iron nanoparticles, allowing for the real-time, non-invasive localization of TBI lesions via fluorescence imaging, SPECT, or MRI.^107^ In addition, AS-Exos can cross the BBB and biodistributed in the brain following intranasal perfusion,^108^ providing an efficient non-invasive delivery route.

## Artificial Intelligence (AI) and Machine Learning (ML) in Nanoparticle-Enhanced Scans

AI and ML techniques are increasingly being integrated into the analysis of complex imaging datasets generated from nanoparticle-enhanced scans in TBI diagnosis.^109^ These computational approaches enable the extraction of high-dimensional features and subtle imaging biomarkers that may be imperceptible to human observers, thereby enhancing diagnostic accuracy. By leveraging large-scale imaging data, AI algorithms can perform automated segmentation of injured brain regions, quantify nanoparticle distribution, and identify patterns correlating with specific pathological processes such as oxidative stress, inflammation, edema, and microhemorrhages. ML models, including convolutional neural networks (CNNs) and support vector machines (SVMs), have been employed to stratify injury severity by categorizing TBI into mild, moderate, and severe classes based on imaging phenotypes enhanced by targeted nanoparticles.^109^ This stratification is critical for tailoring clinical interventions and predicting patient trajectories. Moreover, AI-driven predictive modeling can integrate multimodal data including imaging features, clinical parameters, and molecular biomarkers carried by nanoparticles, to forecast functional outcomes such as cognitive recovery, neurodegeneration risk, or long-term disability.^110^ Additionally, AI facilitates the optimization of nanoparticle design and targeting by simulating biodistribution and pharmacokinetics from imaging data, which may accelerate the development of personalized nanotherapeutics.^111^ Despite these advances, challenges such as data heterogeneity, limited annotated datasets, and the need for model interpretability must be addressed to fully translate AI-enhanced nanoparticle imaging into routine clinical practice.^110^ Future directions involve the incorporation of explainable AI frameworks, federated learning for multi-institutional data sharing, and integration with real-time imaging systems to enable dynamic assessment of TBI progression and response to nanoparticle-based therapies.^111^ The detection of hematomas in TBI using CT is often complicated by variations in their morphology, including differences in size, shape, and anatomical location.^111^,^112^ These complexities can introduce errors in the interpretation of neuroimaging. Moreover, subtle lesions such as microbleeds are frequently overlooked by CNNs, mainly because such findings are underrepresented in existing training datasets. Nevertheless, CNNs hold significant promise for improving diagnostic precision and workflow efficiency. A major challenge lies in their reliance on extensive, high-quality datasets; however, many available CT datasets contain a surplus of irrelevant or non-diagnostic images, which limits model performance. To address this, two-dimensional (2D) montage images have been adopted to streamline data input and reduce manual curation during training. Building on this approach, deep montage-based image retrieval (dMIR) has emerged as a powerful method for reconstructing 3D image contexts from 2D montages, preserving anatomical continuity and enhancing spatial interpretation. Using this technique, a model developed by Kerley et al demonstrated a remarkable accuracy of 98.8% and a precision of 96.2% across 1000 heterogeneous CT scans, with only 12 cases misclassified.^111^,^113^

Examples of the applications of ML to enhance TBI diagnosis include the studies by Rani et al^114^ who fused gold-nanoparticles and quantum-dot-based nanoscale MRI imaging with deep-neural-network analysis to detect brain tumours within minutes, illustrating AI-accelerated interpretation of nanomaterial-enhanced imaging. Sun et al^115^ used silver-nanoparticle-based surface-enhanced Raman spectroscopy (SERS) with ratiometric analysis to intraoperatively quantify glioma cells, while Wang et al.^116^ applied SERS combined with random-forest, polynomial-regression, ANN, SVM and OPLS-DA to cerebrospinal-fluid samples for Alzheimer’s-disease detection, both showing that AI-driven interpretation of nanomaterial-derived spectral data can guide surgical decisions and early diagnosis. Overall, AI accelerates nanomedicine research while enhancing its reliability.

## Therapeutic Applications of Nanoparticles in TBI

**Figure 4:**
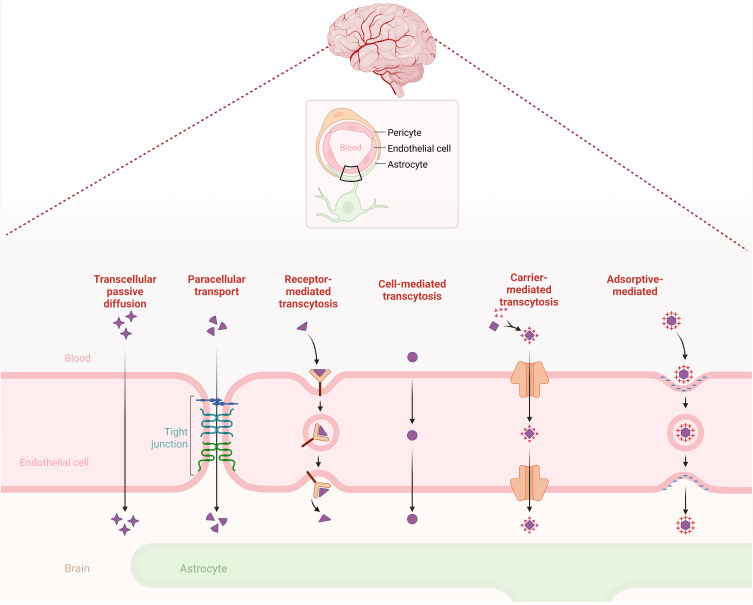
Schematic Overview of Nanoparticle Cargo and Delivery Mechanisms. This figure illustrates the types of cargo and delivery mechanisms used for nanoparticles in biomedical applications. Nanoparticles can be loaded with a variety of therapeutic and diagnostic cargoes, including small drug molecules, peptides, nucleic acids, imaging probes, antioxidants, and stem cell modulators. Delivery can occur via cell-targeted mechanisms, involving specific binding to cell surface receptors, endocytic uptake, and subsequent drug release within lysosomes. Alternatively, stimuli-responsive delivery exploits either external triggers (such as magnetic fields, light, and heat) or internal biological cues (such as pH, redox state, and enzyme activity) to achieve controlled drug release at the target site. This approach enhances specificity and efficacy while minimizing off-target effects.

At the moment, there are no approved pharmaceutical treatments for TBIs, which afflict an extensive range of people. Despite extensive preclinical and early-phase clinical research, numerous pharmacological agents have failed to demonstrate efficacy in large-scale Phase III trials for TBI.^22^,^117–119^ By strengthening the preservation of pharmacological payloads, facilitating more precise medication administration to targeted locations, and possibly integrating with supportive scaffolding to improve secondary outcomes, nanoparticles present a viable solution to this problem (Figure 4).^120^

### CAQK-pSiNPs for BDNF Delivery

In their landmark study, Waggoner et al^121^ engineered porous silicon nanoparticles (pSiNPs) as a systemic delivery vehicle for brain-derived neurotrophic factor (BDNF) to treat TBI in mice. They began by electrochemically etching mesoporous silicon to produce ~130 nm particles with pores ranging from 10 to 20 nm. BDNF was “oxidatively trapped” within these pores at an unprecedented 13 wt% loading, greatly exceeding prior nanoparticle systems (<1 wt%). To prolong circulation and direct the particles to injury sites, the pSiNPs were PEGylated and decorated with the cysteine-alanine-glutamine-lysine (CAQK) peptide a ligand known to bind extracellular matrix components that are upregulated after TBI. In vitro, released BDNF retained full bioactivity, as evidenced by dose-dependent increases in viability and neurite outgrowth in TrkB-expressing SH-SY5Y neurons.

In vivo, CAQK-targeted pSiNPs carrying a fluorescent model protein rapidly accumulated in the injured cortex when injected two hours post-controlled cortical impact, as shown by time-gated silicon photoluminescence and confocal microscopy co-localization of peptide and payload. Critically, a single systemic dose of BDNF-loaded CAQK-pSiNPs reduced lesion volume by 24% at three days post-injury, whereas equivalent free BDNF conferred no benefit, demonstrating that targeting plus sustained release across the transiently permeable BBB markedly enhances therapeutic efficacy.

By combining high loading capacity, injury-site homing, protection of fragile proteins from degradation, and linear 72-hour release kinetics, this pSiNP platform overcomes the key hurdles of BDNF delivery, including its short half-life, poor brain uptake, and non-specific distribution, making it a powerful new advance in TBI therapy.^121^

### CAQK–β-Lactoglobulin/Disulfiram (CAQK–β-LG/DSF) Nanoparticles

These 120 nm particles consist of β-lactoglobulin protein shells loaded with the FDA-approved drug disulfiram (DSF) and decorated on their surface with the CAQK peptide, which binds specifically to chondroitin sulfate proteoglycans upregulated after brain injury. When injected intravenously in a mouse controlled-cortical-impact model, CAQK–β-LG/DSF NPs crossed the transiently disrupted BBB, accumulated selectively at the injury site, and released DSF in response to the local oxidative environment.^122^ There, DSF inhibited gasdermin D–mediated pyroptosis and dampened microglial activation, resulting in a 45% reduction in lesion volume, preservation of neuronal networks, and significant improvements in motor coordination and spatial memory. The experiment was highly successful, with treated animals showing no overt systemic toxicity and durable functional recovery up to 28 days post-injury.^122^

### CAQK Peptide-Modified Antioxidant/Curcumin Nanoparticles (C-PPS/C)

C‐PPS/C provide a dual therapeutic strategy for TBI by simultaneously scavenging ROS and delivering curcumin to the lesion.^123^ In a controlled cortical impact model, a single intravenous dose of ~115 nm C‐PPS/C administered six hours after injury accumulated selectively at the site of damage via the CAQK peptide, which binds chondroitin sulfate proteoglycans exposed in the injured extracellular matrix. The PPS core reacts with hydrogen peroxide, reducing superoxide levels by more than 50%, while the oxidative environment triggers curcumin release. Treatment restored antioxidant enzyme activity specifically glutathione peroxidase (GSH‐Px), which reduces hydrogen peroxide to water, and lowered malondialdehyde (MDA), a lipid peroxidation byproduct and marker of oxidative damage. Preservation of BBB integrity and a shift of microglia toward an anti‐inflammatory phenotype followed, resulting in 35% less brain edema, a 45%reduction in lesion volume, and significant improvements in motor coordination, anxiety‐like behavior, and spatial memory compared to controls.^123^ No systemic toxicity was observed. These findings demonstrate that C‐PPS/C nanoparticles offer a targeted, scientifically grounded approach to mitigate oxidative stress and neuroinflammation following TBI.^123^

### Microglial Exosomes Enriched in miR-124-3p

In the study by Huang et al,^124^ microglial exosomes enriched in miR-124-3p were shown to exert potent anti-inflammatory and neuroregenerative effects in both in vitro and in vivo TBI models. After repeated mild TBI in mice, exosomal miR-124-3p levels rose significantly in microglia, promoting their switch to an M2 (anti-inflammatory) phenotype. When these exosomes were applied to cultured neurons, they inhibited NF-κB– and mTOR-driven inflammatory signaling, reduced the expression of pro-degenerative markers (Aβ-peptide, p-Tau), and increased neurite branching and total neurite length hallmarks of neuronal repair. In vivo, intravenous administration of miR-124-3p-loaded exosomes two hours after controlled cortical impact enabled passage across the BBB, accumulated in the injured cortex, and resulted in a >60% reduction in pro-inflammatory cytokines, preservation of tight-junction proteins (ZO-1, occludin), and markedly improved performance on beam-walk assays at seven days post-injury, compared to controls. Crucially, these benefits were absent when exosomes lacked miR-124-3p, confirming that the loaded microRNA is the key therapeutic cargo.^124^ By dampening microglial-mediated inflammation, stabilizing the neurovascular unit, and directly stimulating neurite outgrowth, MSC-exosome–miR-124 nanocarriers exemplify a multifaceted, targeted approach to mitigate secondary injury cascades in TBI. Their ability to deliver a defined, anti-inflammatory payload while inherently leveraging the natural tropism and biocompatibility of exosomal vesicles positions them as a highly promising nanotherapeutic platform for translation into clinical TBI care.^124^

### PEGylated Multimeric RNA Nanoparticles (PEG-RNPs)

PEG-RNPs offer a novel siRNA therapy for TBI by combining high payload capacity with favorable pharmacokinetics and intrinsic brain-injury tropism.^125^ These 50 nm particles are synthesized via rolling circle transcription to embed siRNA sequences against the pro-inflammatory cytokine TNF-α and are subsequently PEGylated to extend their blood half-life from 10 to 20 minutes.^125^ In vitro, PEG-RNPs achieved over 50% knockdown of TNF-α mRNA in LPS-stimulated BV2 microglia without toxicity. In a mouse-controlled cortical impact model, a single systemic dose administered 2 hours post-injury resulted in a 26% increase in nanoparticle accumulation in the injured hemisphere versus unmodified RNPs, and reduced TNF-α expression by approximately 76% in perilesional tissue at 24 hours. By silencing a key mediator of secondary injury, PEG-RNPs represent a promising RNAi-based approach to reduce neuroinflammation and protect neural tissue after TBI.^125^

### 2-Deoxy Glucose Surfaced Mixed-Layer Dendrimer (2DG‐D)

A study by Dhull et al^126^ describes a novel 2-deoxy-glucose–functionalized mixed‐layer polyamidoamine dendrimer (2DG‐D; 4 nm diameter) engineered for the precise delivery of the PPARγ agonist pioglitazone (Pio) to injured neurons in TBI. By conjugating 2DG to the dendrimer surface, the nanoparticles exploit upregulated GLUT transporters on compromised BBB endothelia and stressed neurons, achieving rapid transcytosis into the injured parenchyma. Following a single intravenous dose administered two hours post–controlled cortical impact, fluorescently labeled 2DG‐D demonstrated preferential accumulation in the perilesional cortex and hippocampus compared to non‐functionalized controls.^126^ Inside neurons, the acidic endosomal environment triggered the release of Pio, which then engaged PPARγ to attenuate NF-κB–mediated pro‐inflammatory signaling. Quantitative analyses revealed a >60% reduction in microglial activation (Iba1⁺ cells) and pro‐inflammatory cytokines (TNF-α, IL-1β), preservation of NeuN⁺ neuronal populations, and restoration of synaptic protein expression (PSD‐95, synaptophysin).^126^ Functionally, 2DG‐D–Pio–treated mice exhibited significant improvements in rotarod endurance and Barnes maze performance at 7- and 14-days post-injury, with no observed off‐target toxicity.^126^

### Astrocyte-Derived Exosomes

In a mouse-controlled cortical impact model, researchers isolated exosomes from healthy astrocyte cultures and then injected them into the bloodstream 30 minutes after injury.^105^ These exosomes crossed the disrupted BBB and accumulated in the damaged cortex, where they released their cargo over several days. Once there, they activated the Nrf2 antioxidant pathway in neurons, impacting enzymes such as superoxide dismutase (SOD) and catalase, while simultaneously suppressing cell-death signals (eg, caspase-3) and limiting inflammation. Functionally, treated mice exhibited a 40% improvement in motor coordination tests, a 30% reduction in lesion size, and markedly better neuronal survival compared to controls.^105^ Crucially, mice lacking the Nrf2 pathway gained no benefit, confirming that exosomal delivery of Nrf2-activating components is central to their neuroprotective effect.

### Intravenous Immunomodulatory Nanoparticles (IMPs)

IMPs are ~500 nm particles made from biodegradable, carboxylated poly(lactic-co-glycolic acid) (PLGA-COOH). In the Sharma et al mouse study,^127^ IMPs were injected into the bloodstream 2 hours after a controlled cortical impact (CCI) or closed-head injury (CHI), and then again at 24 hours and 48 hours. Each IMP binds specifically to MARCO⁺ monocytes (a type of white blood cell), diverting them from the injured brain and trapping them in the spleen. At 72 hours post-injury, treated mice had 84% fewer monocytes/macrophages and 66% fewer total myeloid cells in their brains, with the remaining cells exhibiting an anti-inflammatory (M2-like) profile. By preventing these immune cells from entering, IMPs reduced pro-inflammatory cytokines (TNF-α, IL-1β) by ~50%, preserved BBB tight junctions, and decreased lesion size by 35–45% as observed on MRI. Functionally, IMP-treated mice performed significantly better on the ladder run and rotarod motor tests and exhibited less brain swelling (edema). IMPs were safely cleared by liver and spleen macrophages with no toxicity. This approach, which blocks harmful monocyte entry, effectively dampens neuroinflammation and speeds recovery after TBI.^127^ Pu et al^128^ developed 100 nm lipid–polymer nanoparticles carrying interleukin-4 (IL-4) and injected them intravenously within 30 minutes of TBI. These particles crossed the injured blood–brain barrier, released IL-4 over 48 hours in the oxidative lesion environment, and shifted microglia and macrophages to a healing “M2” state.^128^ Treated mice showed 45% less scarring by astrocytes, 50% fewer activated microglia, preserved white matter integrity on MRI, and a 40% improvement in performance on balance and memory tests, demonstrating a clear neuroprotective benefit without systemic side effects.

### Nanoparticles and Stem Cells Induction

Researchers have begun using nanoparticles to deliver the chemokine stromal-derived factor-1α (SDF-1α) directly into the injured brain, creating a sustained local release that attracts the body’s neural stem cells (NSCs) and neuroblasts toward the lesion.^129^ In a recent mouse study, poly(lactic-co-glycolic acid) (PLGA) nanoparticles carrying SDF-1α were injected around the injury site shortly after trauma.^129^ These particles slowly released SDF-1α over several days, establishing a persistent chemical signal that roughly doubled the number of migrating NSCs reaching the damaged tissue compared to free SDF-1α. As a result, treated animals showed significantly smaller lesion areas and better recovery of motor function.

Narouiepour et al^130^ explored whether curcumin‐loaded niosomes (CM-NPs) could enhance the benefits of transplanted human neural stem cells (hNS/PCs) in a rat model of TBI. Two days after the injury, they grafted stem cells into the area around the damage. Rats that received only stem cells showed some improvement in movement and a reduction in inflammation. However, when the animals were also given CM-NPs, far more of the transplanted cells survived and became mature brain cells, with twice as many new neurons and astrocytes observed compared to stem cells alone. The nanoparticles created a more favorable environment by blocking inflammatory signals and enhancing growth factors such as BDNF and VEGF, which support stem cell growth and integration. As a result, rats treated with both stem cells and CM-NPs performed significantly better on balance and coordination tests than those given only one therapy.^130^ Although still in early preclinical stages, this approach highlights how simple, sustained-release nanoparticles can harness the brain’s intrinsic repair mechanisms to reduce tissue loss after TBI.

## Challenges, Limitations, and Future Directions

### Biological Barriers

Nanoparticles offer a promising avenue for delivering therapeutics to the brain following TBI. However, the BBB presents a significant challenge to their practical use. The BBB is a selective barrier that restricts the passage of substances from the bloodstream into the brain, thereby complicating the delivery of therapeutic agents.^131^

The primary obstacle is the BBB, a selective semipermeable membrane that protects the brain from harmful substances in the bloodstream. Its restrictive nature makes it difficult for therapeutic agents, including nanoparticles, to reach brain tissue. Following TBI, the BBB’s permeability can become unpredictable, complicating the delivery of nanoparticles to the injured site.^132^ Designing nanoparticles capable of crossing the BBB without causing additional disruption is complex. Surface modifications, such as coating with specific ligands, have been explored to enhance brain barrier penetration, but achieving consistent delivery remains challenging.^49^,^133^ Ensuring that nanoparticles selectively target injured brain regions without affecting healthy tissue is another significant challenge. Strategies such as conjugating nanocarriers with antibodies against markers upregulated during inflammation are being investigated to enhance specificity.^134^

TBI can induce alterations in the permeability and function of various biological barriers, leading to unpredictable nanoparticle distribution and clearance rates. This variability complicates the optimization of dosing regimens and therapeutic windows. For instance, TBI can disrupt the blood–cerebrospinal fluid barrier (BCSFB), which regulates the exchange of substances between the blood and cerebrospinal fluid. Such disruption may result in unintended nanoparticle accumulation or inadequate therapeutic concentrations within the CNS.^135^ Additionally, TBI-induced neuroinflammation can activate the mononuclear phagocyte system (MPS), enhancing the clearance of nanoparticles from the bloodstream and reducing their availability for brain targeting. In addition, an elevated immune response necessitates careful consideration of nanoparticle design to prevent premature clearance.^136^ Furthermore, changes in the endothelial glycocalyx layer, a carbohydrate-rich layer lining blood vessels post-TBI, can significantly impact nanoparticle adhesion and penetration, thereby influencing their distribution within the brain.^137^ The glycocalyx serves as a barrier to nanoparticle adhesion and penetration; its degradation could alter the interaction between nanoparticles and the endothelium, potentially affecting the distribution and efficacy of therapeutics.^138^

The properties of nanomaterials for optimal BBB delivery are governed by its engineered physicochemical properties and active surface functionality, but not their core composition (Table 3).^62^,^139^ Organic nanomaterials, such as liposomes and polymeric nanoparticles, are currently the most clinically promising for application in TBI due to their inherent biodegradability and biocompatibility.^140^ The relative ease of modifying their surface facilitates the attachment of targeting ligands (eg, ApoE-mimetic peptides) to leverage RMT, the primary non-invasive transport mechanism.^62^ Although inorganic and carbon-based carriers offer advantages in stability and theranostic applications,^141^ they often face greater regulatory hurdles due to potential toxicity. Therefore, highly functionalized organic carriers optimized for active targeting represent the most advanced platform for CNS delivery.

**Table 3:** Mechanistic Basis of Nanoparticle Efficacy in Traumatic Brain Injury

Feature	Dendrimers (Polymeric NPs)	Exosomes (Astrocyte- or MSC-Derived)	Inorganic Nanoparticles – SPIONs (Fe_3_O_4_/γ-Fe_2_O_3_)	Inorganic Nanoparticles – Ceria (CeO_2_)
Specific TBI Challenge Addressed	Acute neuroinflammation, oxidative stress, and microglial activation during the secondary injury cascade.	Neuronal and glial dysfunction; chronic inflammation; dysregulated intercellular signaling; loss of neuroplasticity.	Real-time diagnosis, monitoring of edema, hematoma, and inflammatory cell infiltration; image-guided drug delivery.	Acute oxidative burst, mitochondrial dysfunction, and lipid peroxidation after severe/focal injury.
Physicochemical Advantage	Highly branched, monodisperse polymer with tunable size (5–20 nm); high surface area and multivalent loading; easy surface functionalization.	Nano-vesicular bilayer (30–150 nm) carrying miRNAs, proteins, and lipids; inherently biocompatible and low-immunogenic; native receptor-mediated CNS tropism.^142^	Magnetic iron-oxide core provides superparamagnetic behavior; adjustable coating (PEG, dextran) for stability and targeting.	Crystalline Ce^3^⁺/Ce^4^⁺ lattice enabling continuous redox cycling; high structural stability; surface easily PEGylated or citrate-coated.
Mechanistic Pathways Underlying Superior Efficacy	• Anti-inflammatory signaling: suppresses microglial NF-κB and cytokines (TNF-α, IL-1β).^143^ • ROS scavenging via conjugated antioxidants (NAC, melatonin) or intrinsic quenching.^144^ • BBB repair through tight-junction modulation. • Neuroprotection via mitochondrial stabilization and reduced excitotoxicity.	• Anti-inflammatory signaling via miRNAs (miR-124, miR-146a) inhibiting NF-κB/NLRP3.^145^ • ROS scavenging indirectly through Nrf2-HO-1 activation.^105^ • BBB repair through astrocyte–endothelial signaling. • Neuroregeneration via BDNF, GJA1-20k-mediated plasticity.^146^	• ROS scavenging through Fe-dependent Fenton-like modulation. • Imaging contrast for MRI-based lesion visualization. • Macrophage polarization toward M2 phenotype. • BBB integrity monitoring by MRI tracking.^147^	• Catalytic ROS scavenging through Ce^3^⁺/Ce^4^⁺ redox cycling.^148^• Mitochondrial protection and ATP preservation. • Anti-inflammatory effects via NF-κB and iNOS inhibition.^149^ • BBB repair through endothelial oxidative stress reduction.
Preferred TBI Model Application	Acute or diffuse TBI models targeting cytokine storm and oxidative injury (within 24–72 h); mild–moderate severity.	Subacute-to-chronic TBI models focused on neurorepair, axonal remodeling, and functional recovery.	Focal contusion models requiring longitudinal imaging and guided delivery.	Severe focal/hemorrhagic TBI models dominated by oxidative stress and mitochondrial dysfunction.
Delivery Route & Targeting	Intravenous or passive (size-based) and active ligand-mediated targeting (transferrin, folate, peptides).^150^	Intravenous or natural targeting enhanced by engineering (RVG, Lamp2b, CXCR4 peptides).^151^	Intravenous; passive accumulation in disrupted vasculature; optionally magnetically guided.^152^	Intravenous; passive targeting of lesion margins.
Primary Limitation & Optimization Need	Surface-charge-dependent cytotoxicity; requires PEGylation or charge neutralization to minimize RES clearance and toxicity.	Rapid systemic clearance and variable BBB penetration; requires surface modification or optimized donor-cell source.	MRI signal blooming and long-term iron persistence; need for biodegradable coatings.	Non-biodegradable core; possible accumulation; requires ultrasmall size and safe coating to mitigate long-term toxicity.

**Notes**: Table 3. Summary of key mechanisms, advantages, and limitations of major nanoparticle classes used in TBI therapy and imaging.

The long-term effects of nanoparticles on brain health are not fully understood. There is a risk that nanoparticles may induce neuroinflammation or other adverse reactions, necessitating thorough biocompatibility assessments.^153^ Strategies such as conjugating nanocarriers with antibodies against markers upregulated during inflammation are being investigated to enhance specificity.^154^ Moreover, advancements in nanoparticle-based therapies for TBI have shown promise in overcoming the challenges posed by the BBB. Recent studies have developed nanoparticles capable of crossing the BBB to deliver therapeutic agents directly to the brain. For instance, researchers engineered nanoparticles to carry small interfering RNA (siRNA) molecules designed to inhibit tau protein production, demonstrating effective delivery to the brain and a reduction in tau levels in a mouse model of TBI.^155^ However, translating these promising therapies into clinical practice involves overcoming substantial challenges, including navigating complex regulatory pathways, ensuring consistency in large-scale manufacturing, and conducting rigorous clinical trials to establish their safety and efficacy.^156^

### Safety and Toxicity Concerns

Nanoparticle-based therapies hold promises for treating TBI; however, safety and toxicity concerns must be carefully addressed. The unique properties of nanoparticles, such as their size, shape, and surface characteristics, can lead to unforeseen interactions within biological systems. For instance, specific nanoparticles may induce oxidative stress, inflammation, or cytotoxicity, potentially exacerbating neural damage.^157^ Additionally, the long-term fate of nanoparticles in the body remains uncertain, raising concerns about their accumulation and potential for chronic toxicity.^158^

The interaction of nanoparticles with biological systems is complex and influenced by various factors. For example, the formation of a protein corona on the nanoparticle surface can alter its biological identity, affecting cellular uptake and toxicity. Studies have shown that nanoparticles can elicit a wide range of intracellular responses, depending on their properties, concentrations, and interactions with biological molecules. These properties and their relationships to cellular function can induce cellular damage or advantageous cellular responses, such as increased energy production and growth.^159^ Moreover, the biodistribution and accumulation of nanoparticles in organs over time can lead to chronic inflammation. Soluble nanoparticle components, such as metal ions, also drive toxicity through oxidative damage, protein binding, enzyme inhibition, and other mechanisms.^36^,^160^

Recent studies have explored various nanoparticle formulations to mitigate these safety concerns. For example, neutrophil membrane-derived nanoparticles have been investigated for their ability to inhibit calcium overload and scavenge reactive oxygen species, thereby protecting against secondary injury in TBI models.^161^ Additionally, dendrimer-based nanoparticles have shown promise in targeting neuroinflammation. Hydroxyl-terminated polyamidoamine (PAMAM) dendrimers can cross the blood-brain barrier and localize within activated microglia and astrocytes, delivering therapeutic agents directly to sites of inflammation. This targeted approach has demonstrated efficacy in reducing neuroinflammation and improving functional outcomes in animal models of TBI.^162^

Given these complexities, it is imperative to conduct thorough in vitro and in vivo studies to assess the potential risks associated with nanoparticle-based therapies for TBI.^163^ This includes evaluating the impact of nanoparticle physicochemical properties on their interaction with biological systems, understanding the mechanisms underlying nanoparticle-induced toxicity, and developing strategies to mitigate adverse effects. By addressing these challenges, researchers can improve the safety and efficacy of nanoparticle-based treatments for TBI.^164^

Long-term safety data for repeated CNS NP dosing indicate that most NPs provoke only a transient microglial response that resolves when the particles are cleared, but chronic exposure can shift microglia toward a sustained pro-inflammatory phenotype and promote delayed neurotoxicity. In a developing mouse model, microglia rapidly phagocytosed silica and polystyrene NPs via a complement-C3–dependent pathway, and this acute clearance protected neurons; however, blocking the chemokine-CCR4 axis that drives C3 production impaired phagocytosis, increased neuronal loss and produced anxiety-like behavior, highlighting that efficient immune clearance is essential for safety.^165^ Studies of metal-based NPs show that they can remodel microglial dynamics and, if not properly engineered, generate reactive-oxygen-species–rich environments that exacerbate neuroinflammation and impede regeneration, suggesting a risk of chronic oxidative stress.^166^ Conversely, amphiphilic NPs designed to target scavenger receptors attenuated Aβ-induced microglial activation, reduced pro-inflammatory cytokine release, and prevented downstream neurotoxicity in vitro, providing a proof-of-concept that NP surface chemistry can suppress chronic microglial activation.^167^ The broader nano-bio interface literature warns that persistent activation of microglia and astrocytes can maintain a cycle of cytokine and ROS production, which over time contributes to neuronal damage in diseases such as Alzheimer’s and multiple sclerosis. Thus, preclinical evidence underscores two key safety principles: (1) NPs must be engineered for rapid, complement-mediated clearance to avoid prolonged microglial engagement, and (2) surface functionalization should actively dampen pro-inflammatory signaling to prevent chronic neuroinflammation and delayed neurotoxicity.

### Regulatory Challenges and Clinical Trials for Nanoparticle-Based Therapies

Regulatory challenges for nanoparticle-based therapies in TBI are multifaceted and complex. One of the primary challenges is the need for comprehensive regulatory guidelines to assess the safety, efficacy, and biocompatibility of nanoparticles.^168^,^169^ Unlike traditional drug formulations, nanoparticles possess unique physicochemical properties, such as size, shape, surface charge, and surface modifications, which can significantly influence their behavior in biological systems.^168^ Regulatory bodies require comprehensive preclinical and clinical data to evaluate the safety, efficacy, and biocompatibility of nanoparticles. This necessitates rigorous testing across various animal models to assess biodistribution, toxicity, and long-term effects.^156^ Moreover, the manufacturing of nanoparticles at scale poses additional hurdles. Ensuring batch-to-batch consistency, reproducibility of nanoparticle characteristics, and maintaining high-quality control standards are crucial to meeting regulatory requirements.^170^ Furthermore, the dynamic nature of nanoparticle interactions within the body and the potential for unforeseen immune responses complicate the approval process.^171^ As a result, regulators must balance the innovative potential of nanoparticle therapies with thorough safety assessments, which often leads to lengthy approval timelines and high costs for clinical translation.^169^ Addressing these regulatory challenges will be crucial to the successful integration of nanoparticle-based therapies into clinical practice for treating TBI.

Despite clear efficacy in preclinical TBI models, no clinical trials of nanoparticle-mediated drug delivery have yet been initiated in TBI patients, underscoring a substantial translational gap.^172^ In contrast, nanoparticle-based platforms are already advancing in human CNS applications for example, the Phase II evaluation of the gadolinium-based nanoparticle AGuIX in brain metastases (NCT04899908)^173^ and the Phase II intranasal nanoparticle formulation APH-1105 for Alzheimer’s disease (NCT03806478).^174^ This disparity highlights a critical need for focused translational initiatives to advance nanoparticle-based applications into clinical testing in TBI.

### Scalability and Cost of Nanoparticle Production

The scalability and cost-effectiveness of nanoparticle production for treating TBI are significant considerations in advancing these therapies from laboratory research to clinical application. Manufacturing nanoparticles at a scale suitable for clinical use requires the development of standardized, reproducible processes that maintain the desired physicochemical properties of the nanoparticles.^175^ This includes ensuring consistent size, surface charge, and functionalization, which are critical for their efficacy and safety. Achieving such consistency often necessitates sophisticated equipment and stringent quality control measures, contributing to increased production costs.^176^ Additionally, the materials used in nanoparticle synthesis can influence both the cost and scalability. While some materials are readily available and cost-effective, others may be expensive or require complex synthesis methods, which can impact the overall production cost. For instance, biodegradable polymers like poly(lactic-co-glycolic acid) (PLGA) are commonly used due to their biocompatibility and controlled release properties. However, the synthesis of PLGA nanoparticles requires precise control over polymerization conditions, which can be resource-intensive and costly.^177^

Furthermore, the regulatory requirements for nanoparticle-based therapies add another layer of complexity. Manufacturers must comply with stringent guidelines to ensure the safety and efficacy of the nanoparticles, which can involve extensive preclinical and clinical testing. These regulatory processes are time-consuming and expensive, further increasing the overall cost of developing nanoparticle-based treatments for TBI.^178^

Future directions should focus on developing cost-effective manufacturing processes and materials, along with navigating regulatory requirements, to facilitate the successful translation of these therapies into clinical practice. Adopting green synthesis methods can contribute to more sustainable and cost-effective production. Utilizing biological agents, such as plant extracts or microorganisms, for nanoparticle synthesis offers an eco-friendly alternative to traditional chemical methods, potentially reducing production costs and environmental impact.^179^ Furthermore, developing biodegradable and eco-friendly nanoparticles can address environmental concerns and reduce production costs. By focusing on sustainable materials and processes, the overall cost of nanoparticle production can be reduced, making these therapies more accessible for clinical applications.^180^

## Conclusion and Prospects

Nanotechnology is reshaping TBI diagnostics and therapeutics by enabling precise imaging, targeted drug delivery, controlled release, and modulation of neuroinflammation. Organic systems (dendrimers, polymeric NPs, lipids, exosomes) and inorganic platforms (iron oxide, gold, quantum dots) offer unique strengths in crossing the BBB, enhancing lesion visualization, and delivering neuroprotective or anti-inflammatory agents. When combined with AI and machine learning, nanoparticle-based imaging and sensing gain additional power improving lesion detection, quantifying molecular changes, and enabling more accurate patient stratification.

Despite these advances, key challenges remain. Biological barriers, variability in nanoparticle formulations, and incomplete understanding of long-term biodistribution and neurotoxicity limit translation. Heterogeneity in exosome preparations, protein-corona effects, and difficulty achieving scalable, reproducible manufacturing also present obstacles. Regulatory pathways for nano-enabled diagnostics and therapeutics are still evolving, particularly regarding safety, batch consistency, and environmental impacts.

## Data Availability

Not Applicable. This is a review article, and all relevant information is provided within the article.
